# Glutamate dehydrogenase 1 mediated glutaminolysis sustains HCC cells survival under glucose deprivation

**DOI:** 10.7150/jca.64195

**Published:** 2022-01-04

**Authors:** Yujiao Zhou, Haibo Yu, Shengtao Cheng, Yao Chen, Lin He, Jihua Ren, Xin He, Juan Chen, Lu Zheng, Fan Li

**Affiliations:** 1 The Key Laboratory of Molecular Biology of Infectious Diseases designated by the Chinese Ministry of Education, Chongqing Medical University, Chongqing, 400016, China; 2 Department of Medical examination center, The second Affiliated Hospital of Chongqing Medical University, Chongqing, China; 3 Department of Hepatobiliary Surgery, the Second Affiliated Hospital of Army Medical University, Chongqing, 400037, China; 4 Department of Endocrine and Breast Surgery, The First Affiliated Hospital of Chongqing Medical University, Chongqing, China

**Keywords:** HCC, Glutaminolysis, GDH1, TCA cycle

## Abstract

Besides aerobic glycolysis, glutaminolysis has also become a hot spot in the field of tumor research because of its important role in regulating cell proliferation, apoptosis, and migration and invasion. Meanwhile, it is generally believed that tumor cells could sustain its proliferation and survival according to a so-called metabolic flexibility. How the metabolic flexibility of HCC cells behaves has not yet been fully elucidated. In this study, we validated the glutamine addiction of HCC cells, and identified that the glutaminolysis pathway of HCC cells altered in response to different glucose conditions. That is, glutamate transaminases GOT1 pathway played a dominant role in regulating cell growth when glucose was sufficient, yet deaminase GDH1 mediated metabolic pathway became dominant when glucose was limited, for the reason that GDH1 could drive the TCA cycle in response to glucose deprivation. Additionally, we further uncovered an negative relationship between GDH1 and GOT1 in low-glucose HCC tissues. Together, our study provided a new insight into the metabolic flexibility of glutaminolysis related enzymes in HCC, and highlighted the crucial role of GDH1 on HCC cells proliferation and survival in glucose starvation.

## Introduction

Metabolic reprogramming is a hallmark of cancer cells and plays a critical role in tumor cell survival, proliferation and migration and invasion 1. Hepatocellular carcinoma (HCC) is one of the most common malignant tumors in China, which is characterized by insidious onset, easy metastasis, a low 5-year survival rate, and lack of diagnostic techniques to identify early symptoms. The occurrence and progression of HCC are also closely intertwined with its metabolic disorders. Similar to most other solid tumors, the metabolic process of HCC cells has undergone many significant changes compared with normal liver cells, which are manifested in abnormally elevated glycolytic activity, increased de novo synthesis of fatty acids and decreased oxidation, as well as accelerated glutamine catabolism 2-4.

Glutamine is the most abundant circulating amino acid in blood and muscle, and participates many fundamental cell functions in cancer cells, including generation of antioxidants to remove reactive oxygen species; synthesis of biological macromolecules; and activation of cell signaling. Glutaminolysis is the process by which cells convert glutamine into tricarboxylic acid (TCA) cycle metabolites through the activity of a series of enzymes. Firstly, glutamine is converted into glutamate by glutaminase (GLS). Subsequently, glutamate is converted into α-ketoglutarate (α-KG) via two divergent pathways. One way is via activation of glutamate dehydrogenase (GDH), and the other is achieved by activation of a group of transaminases, which includes glutamate-oxaloacetate transaminase (GOT), glutamate-pyruvate transaminase (GPT), and phosphoserine transaminase (PSAT). Liver cancer has a metabolic dependency on glutamine 5, indicating an essential role of glutamine enzymes mentioned above in the occurrence and development of HCC. A study by Li et al. reported that GLS1 was highly expressed in liver cancer and regulated stemness properties of HCC cells via ROS/Wnt/β-catenin signaling 6. However, levels of other crucial glutamine enzymes and its regulatory effect on HCC cells remain to be fully elucidated.

It is widely accepted that cancer cells usually optimize nutrient utilization when resources are scarce. Such adaptive mechanism is defined as metabolic flexibility 7,8. One previous study found that glucose deprivation resulted in the emergence of a selective pressure for KRAS mutation in colon cancer cells, thus, the mutated KRAS rendered cells tolerant of low glucose conditions 9. Another research uncovered that expression changes of some glutamine enzymes could lead to a pronounced suppression of cell growth in pancreatic cancer 10. Nevertheless, how does metabolic flexibility manifest in HCC is not fully understood. In this study, we reported the addiction of HCC cells to glutamine, and we identified, for the first time, that under different glucose concentration levels, the major glutaminolysis pathway could change in HCC. That is, GOT1 mediated pathway played a dominant role in regulating cell growth when glucose was sufficient, yet GDH1 regulated deamination reaction was necessary for cell survival when glucose was limited, there was an underlying negative correlation between GDH1 and GOT1 in HCC. Mechanically, we found that GDH1 could drive the tricarboxylic acid (TCA) cycle to maintain cell proliferation in response to glucose starvation.

## Materials and Methods

### HCC specimens and cell lines

Tumorous liver tissues and the corresponding adjacent nontumoral liver tissues were collected from 30 patients who underwent curative surgery for HCC at the First Affiliated Hospital of Chongqing Medical University in Southwest China. The patients were not subjected to any form of chemotherapy prior to surgery. Informed consent was obtained from each patient recruited, and the study protocol was approved by the Clinical Research Ethics Committee of Chongqing Medical University.

Normal liver cell line L02, two HCC cell lines SK-Hep-1 and PLC/PRF/5 and breast cancer cell line MDA-MB-231, authenticated by short-tandem repeat (STR) fingerprinting by Beijing Micro-read Genetics Company recently, were cultured in DMEM (Gibco) containing 10% fetal bovine serum (FBS, Hyclone) at 37℃ in 5% CO2. Indicated cells were further maintained in different glucose concentration.

### Small interfering RNAs and antibodies

Small interfering RNAs (siRNAs) were purchased from Shanghai Genechem Company Limited. Targeting sequence of all siRNAs were listed in Supplementary Table S1. GOT1 expression vector (#HG14196-CM) were obtained from Sino Biological Inc. (Beijing, China). Anti-GDH1 (#ab89967), Anti-GOT1 (#ab170950) were obtained from Abcam Inc. (MA, USA) and Anti-β-actin (#9027) were purchased from Cell Signaling Technology Inc. (BOS, USA). Anti-GAPDH (sc-365062) were obtained from Santa Cruz Biotechnology (TX, USA).

### Western blot analysis

Cells were harvested and lysed with RIPA lysis buffer supplemented with a protease inhibitor cocktail (Roche Diagnostics, Indianapolis, IN, USA). Lysate proteins were separated using 10% sodium dodecylsulfate-polyacrylamide gel electrophoresis and transferred to polyvinylidene difluoride membranes (GE Healthcare, Little Chalfont, UK). After incubation with primary antibody overnight, the membranes were incubated with anti-rabbit or anti-mouse IgG secondary antibodies. The blots were developed with ECL Western blot reagents (Millipore, Billerica, MA, USA). The signal intensities were quantified using Image J software.

### Quantitative real-time PCR (qRT-PCR) analysis

Total RNA was prepared using TRNzol Reagent (Tiangen Biotech Co. Ltd., Beijing, China). Relative quantification of gene expression was conducted using FastStart Universal SYBR Green Master Mix (Roche Diagnostics, IN, USA) with β-actin mRNA as an endogenous control. The sequences of primers used in this study were listed in Supplementary Table S2. The expression values of target genes were calculated using 2^-△△Ct method.

### Cell proliferation assay and colony formation assay

Cell proliferation was determined by trypan blue exclusion assay (Thermo Fisher Scientific, Waltham, MA, USA). To investigate the effect of glutamine on cell growth, culture medium were supplemented with 2 mM L-glutamine (Gibco, 210-51-024), 4mM dimethyl-2-oxoglutarate (α-KG, Sigma-Alsdrich, 349631) and 2 mM nonessential amino acid (NEAA, Gibco, 11140), respectively. In addition, enzyme inhibitor epigallocatechin gallate (EGCG, selleckchem, S2250) at concentration of 0, 25, 50 and 100 μM and another enzyme inhibitor aminooxyacetate (AOA, selleckchem, S4989) at concentration of 0, 0.5, 1.0 and 2.0 mM were added to culture media, respectively, aiming to observe their effects on HCC cell growth.

For colony formation assay, cells were grown in puromycin-containing medium (1.0 μg/mL) for 10 days. Finally formed colonies were stained with 0.5% crystal violet.

### CCK-8 assay

2000 cells/well were seeded in a 96-well culture plate. Cell viability was assayed by Cell Counting Kit-8 (CCK-8) for 4 days continuously with a 1-day interval. Working solution (MedChemExpress, #HY-K0301) was added to cells at indicated times and the mixture was incubated for 3 h. The optical density (OD) values were measured with a microplate reader (Bio-Tek) at 450 nm. Cell viability was expressed as a percentage of the maximum absorbance from three replicates in three independent experiments.

### Apoptosis assay

The HCC cells were treated with different concentrations of EGCG or AOA mentioned above and cultivated for 48 h. Cells were firstly stained using Annexin V-FITC Apoptosis Detection Kit (Beyotime Biotechnology) according to the manufacturer's instruction. Then, the percentage of apoptosis cells was tested by flow cytometry (BD Accuri C6).

### Glutamine and ammonium measurements

The amount of glutamine and ammonia in cells and supernatant were measured by L-glutamine / ammonia (rapid) assay kit from Megazyme Inc. (Wicklow, Ireland). All values were normalized to cell numbers done in parallel.

### Glucose assessment

The glucose levels in tissues were detected using glucose assay kit (Abcam ab65333). Tissues were dissolved in double distilled water and homogenized by ultrasonic treatment. A PCA/KOH deproteinization step was performed before glucose assay. All the steps were conducted as the protocol described.

### Energy metabolite analysis

Metabolomic analyses were performed by multiple reaction monitoring (MRM) method. Samples were stable GDH1-silenced PLC/PRF/5 cells and negative control cells cultivated for 3days in medium containing 1.0mM glucose, as well as negative control cells cultivated for 3days in medium containing 25mM glucose. After ultrasound treatment, reconstitution with 1:1 (v/v) acetonitrile/water solution and centrifugation, the supernatant were used for mass spectrometry detection. Heatmaps were constructed based on the results of the metabolite levels.

### Statistical analysis

Data were expressed as means ± SD from at least three independent experiments. All statistical analyses were performed using GraphPad Prism 8 software (GraphPad Software) and SPSS version 19.0 software (SPSS, Inc., Chicago, IL). Comparisons between two groups were performed using Mann-Whitney test. One way ANOVA followed by Dunnett's post-hoc test was performed for comparisons among multiple groups. The value of P<0.05 was considered statistically significant.

## Results

### HCC cells were addicted to glutamine

**Fig 1:**
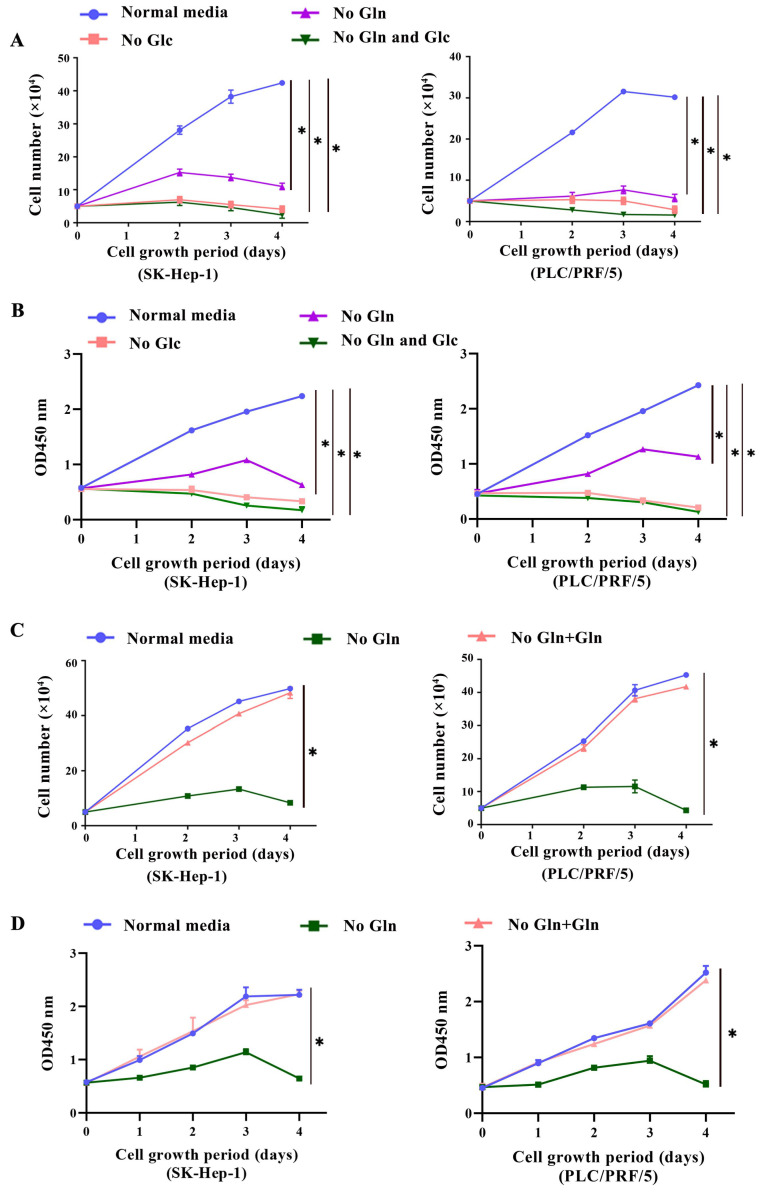
** HCC cells were addicted to glutamine. a-b,** SK-Hep-1 and PLC/PRF/5 cells were cultured in normal medium (Normal media group), normal medium containing no glutamine (No Gln group) or no glucose (No Glc group), respectively. Cells was counted at indicated time points using a trypan blue exclusion assay **(a)** and the CCK-8 assay **(b)**, respectively. *P < 0.05, when compared to that in Normal media group. **c-d**, In addition, SK-Hep-1 and PLC/PRF/5 cells were cultured in medium containing 2mM glutamine (No Gln + Gln group). The proliferation of cells was calculated by the trypan blue exclusion assay (**c**) and the CCK-8 assay (**d**), respectively. *P < 0.05, when compared to that in No Gln group.

To evaluate the glutamine addiction of HCC cells, two HCC cell lines SK-Hep-1 and PLC/PRF/5 were cultured in normal medium, medium without glutamine and medium without glucose, respectively. The proliferate abilities of cells were conducted by trypan blue exclusion method and CCK-8 assay. Meanwhile, normal liver cell line L02, which has no glutamine addiction^11^, as well as breast cancer cell line MDA-MB-231, which has been reported to be glutamine addictive^12^, were cultured under the same conditions and served as controls, respectively. Firstly, we found that starvation of glucose in HCC cells resulted in growth arrest and cell death. In parallel, a marked reduction in cell proliferation was also observed in glutamine-deprived condition. The cell growth was almost arrested after 2 days of glutamine deprivation (Fig. 1A, 1B), indicating the glutamine dependency of HCC cells. Then, we discovered that adding additional 2 mM glutamine was able to support the growth of HCC cells in glutamine-free conditions (Fig. 1C, 1D), indicating the glutamine dependency of HCC cells. AS expected, we did not discover the glutamine addiction in L02 cell (Supplementary Fig. S1A), whereas MDA-MB-231 was depended on glutamine (Supplementary Fig. S1B). Furthermore, we uncovered that the growth arrest induced by glutamine deprivation could be remarkably attenuated by exogenously addition of 0.1 mM NEAAs along with 4mM α-KG, two major intermediates of glutaminolysis. However, adding additional α-KG or NEAAs alone couldn't serve the same role (Supplementary Fig. S1C, S1D). Together, these results indicated that glutaminolysis and its downstream metabolites were necessary for the growth of HCC cells.

### Major glutaminolysis pathway of HCC cells changed under different glucose concentration levels

**Fig 2:**
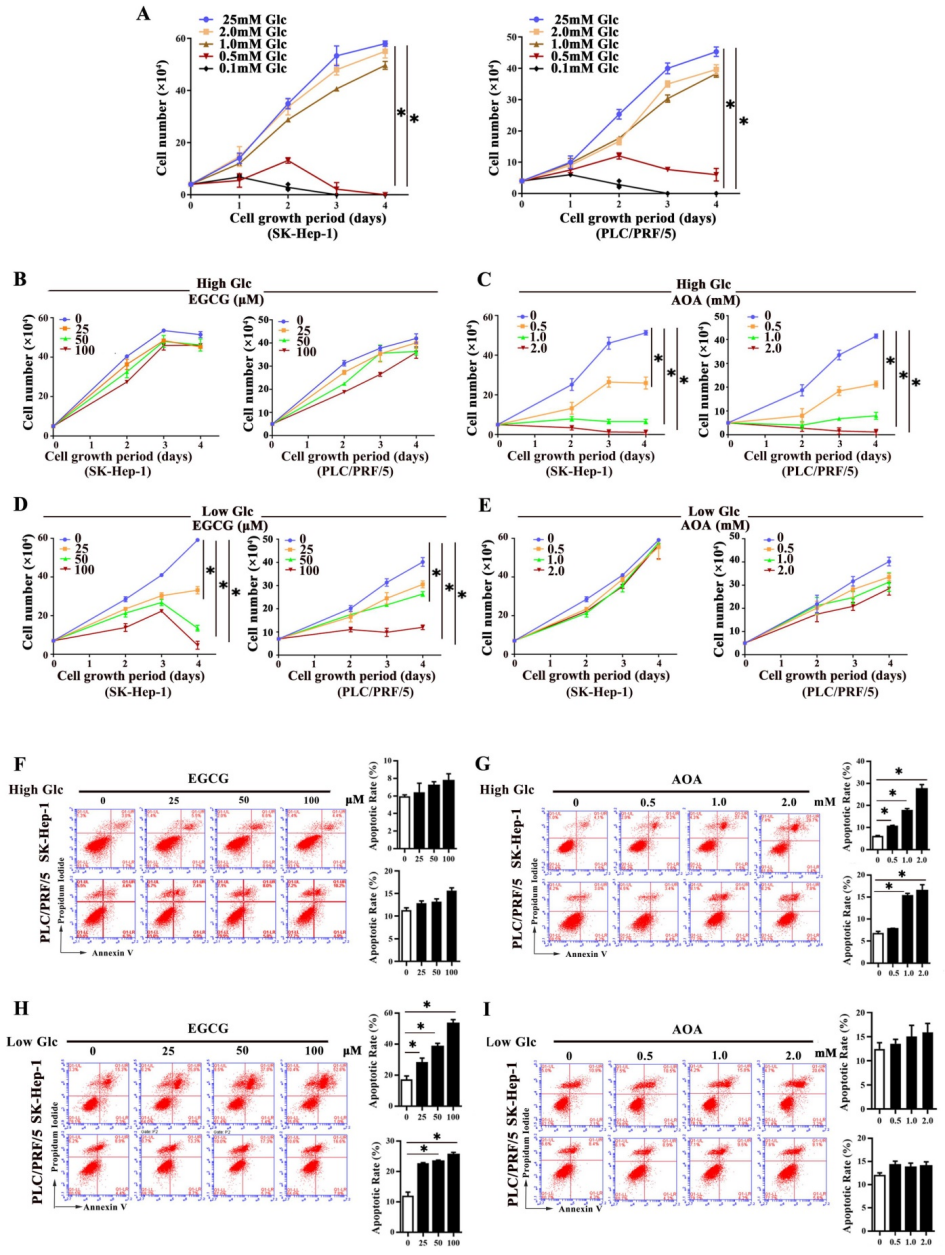
**Major glutaminolysis pathway of HCC cells changed under different glucose concentration levels. a,** The proliferation of SK-Hep-1 and PLC/PRF/5 cells in complete medium containing 0.1mM, 0.5mM, 1.0mM, 2.0mM or 25mM glucose, respectively, was assessed by a trypan blue exclusion assay. *P < 0.05, vs. 25mM glucose. **b-e**, SK-Hep-1 and PLC/PRF/5 cells were exposed to various concentrations of epigallocatechin gallate (EGCG) (**b**) and aminooxyacetate (AOA) (**c**) in high-glucose (25mM) conditions for 72 h, respectively. Similarly, cells were exposed to EGCG (**d**) and AOA (**e**) respectively and cultured in low-glucose (1.0 mM) conditions for 72 h. The cell numbers were obtained. *P < 0.05, vs. 0 group. **f-i,** The apoptosis was analyzed by flow cytometer with Annexin V/PI after treatment with EGCG (**f**) and AOA (**g**) under high glucose conditions for three days, respectively. *P < 0.05, vs. 0 group. Similarly, the effect of various concentrations of EGCG (**h**) and AOA (**i**) on cells apoptosis was analyzed in low glucose media, respectively. *P < 0.05, vs. 0 group. Results were expressed as the average of three independent experiments (n=3 per group).

To investigate whether glutaminolysis pathway changed in response to different glucose levels, HCC cells were cultivated in high glucose medium (25mM glucose) and low glucose medium (1.0 mM glucose), respectively. We defined medium contains 1.0 mM glucose as low glucose culture for the reason that HCC cells in media containing glucose less than 1.0mM could barely survive (Fig. 2A). As shown in Fig. 2B, 2C, and supplementary Fig. S2A, under high glucose concentration, even in cells exposed to 100 μM EGCG, an inhibitor of GDH1 13, no significant inhibition of growth was seen in HCC cells. However, under the same conditions, obviously inhibition of cell growth was observed when cells were exposed to a low concentration of AOA (0.5 mM), a pan-inhibitor of transaminases 14, indicating that cell growth was more dependent on the activities of transaminases under high glucose conditions. Conversely, EGCG intervention markedly suppressed cell growth under low glucose conditions, yet AOA intervention didn´t produce any remarkable anti-growth effect on HCC cells in the same conditions (Fig. 2D, 2E, and Supplementary Fig. S2B), suggesting that GDH1 mediated glutaminolysis played a more key role on cell growth in response to low glucose conditions. Additionally, the roles of EGCG or AOA treatment on cell apoptosis were also detected. As indicated in Fig 2F to 2I, and supplementary Fig. S2C, S2D, AOA treatment robustly promoted cell apoptosis in high glucose conditions, while EGCG intervention markedly induced apoptosis in low glucose conditions. Moreover, under the same culture conditions, we also observed the same changes in cell proliferation and apoptosis in MDA-MB-231 cells (Supplementary Fig. S3A and S3B). Our data preliminary found that glutaminolysis on HCC cells existed a metabolic flexibility in response to altered glucose levels, and such metabolic flexibility of glutaminolysis might be prevalent in cancer cells addictive to glutamine.

To further identify the specific transaminase(s) involved in glutaminolysis, we impaired the activity of metabolic enzymes by individual gene silencing. The expression of indicated genes were successfully inhibited (Supplementary Fig. S4A). Consistent with the previous results, interference of GOT1 significantly weakened HCC cells proliferation under high glucose conditions, while silencing of GDH1 did not take effect (Supplementary Fig. S4B and S4C). Furthermore, we also found that deletion of GDH1 significantly suppressed cells proliferation under glucose limitation, yet knock-down of GOT1 did not take effect (Supplementary Fig. S5A and S5B). Surprisingly, inhibiting glutamine-dependent transaminases including GOT2, GPT-1, GPT-2, PSAT-1 had essentially no impact on proliferation of cells no matter under high or low glucose conditions (Supplementary Fig. S4D, Supplementary Fig. S5C).

**Fig 3:**
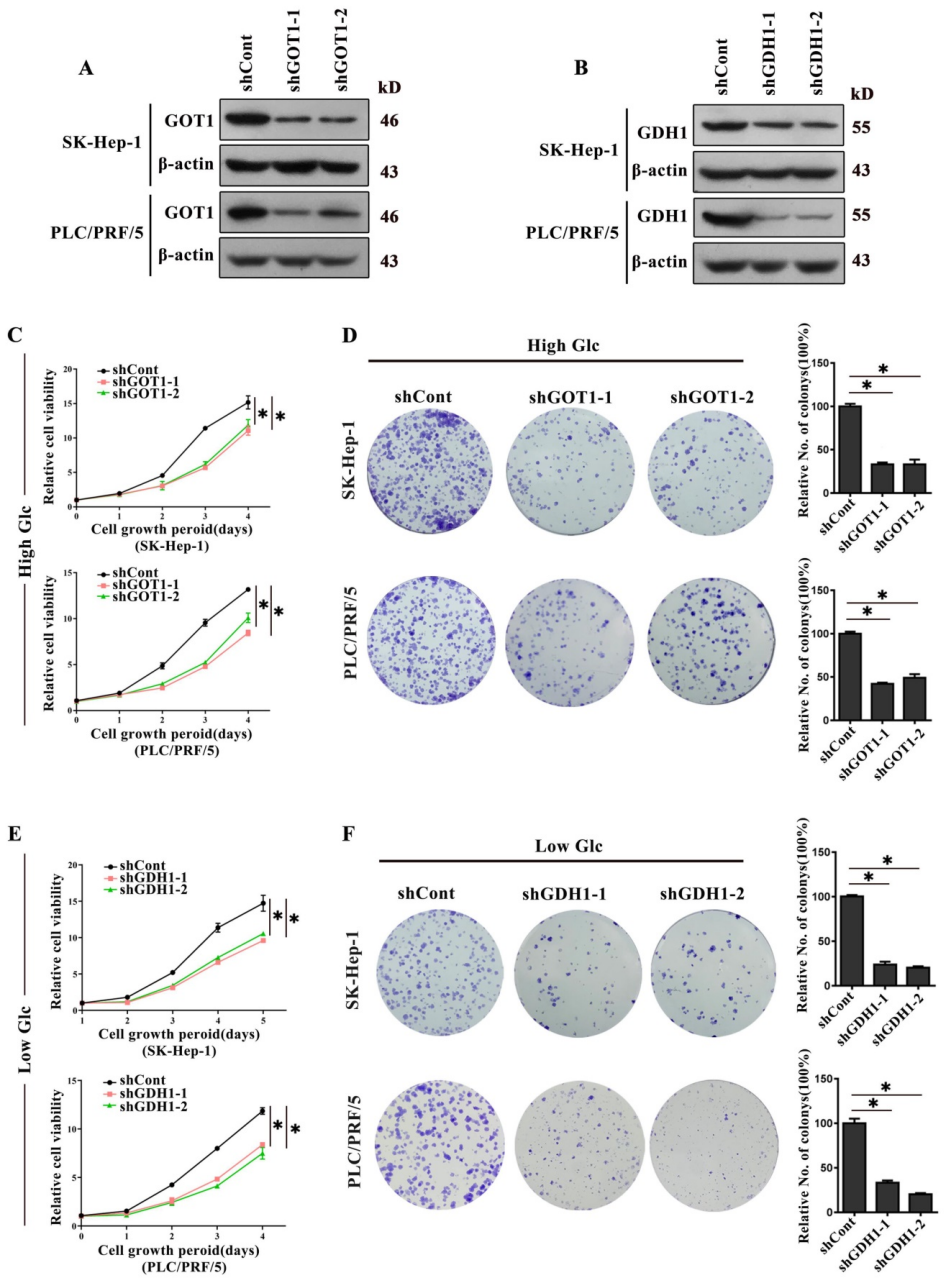
** Glutaminolysis related enzymes served different roles in response to different glucose conditions. a-b**, The GOT1 protein levels (**a**) and GDH1 protein levels (**b**) in SK-Hep-1 and PLC/PRF/5 cells transfected with shRNAs were detected by Western blot. β-actin was used as an internal control. (**c**) The effect of GOT1 knockdown on cell viability in high glucose (25mM) medium was detected by CCK-8 assay. *P < 0.05, vs. shCont. **d,** The effect of GOT1 knockdown on cells colonies formation viability in high glucose (25mM) medium was detected by colony formation assay. Newly formed colonies in each well were counted and expressed as a percentage relative to the control group. *P < 0.05, vs. shCont. **e**, The effect of GDH1 deficiency on cell viability in low glucose (1.0mM) medium was detected by CCK-8 assay. *P < 0.05, vs. shCont. **f**, The efficacy of silencing GOT1 in colony-formation viability of HCC cells in low glucose (1.0mM) medium was detected by colony formation assay. Newly formed colonies in each well were counted and expressed as a percentage relative to the control group. *P < 0.05, vs. shCont. Results were expressed as the average of three independent experiments (n=3 per group).

Additionally, stable knockout cell models were generated by lentivirus expressing an shRNA targeting GOT1 and GDH1, respectively (Fig. 3A and 3B). As expected, GOT1 knock-down remarkably decreased the proliferation and colonies formation when HCC cells were cultivated in medium containing high glucose (Fig. 3C and 3D). In contrast, GDH1 depletion remarkably reduced the proliferation and colonies formation when HCC cells were cultivated in low glucose medium (Fig. 3E and 3F). Collectively, all the data showed that GOT1 catalyzed glutamine transamino-metabolism played a major role on HCC cells survival when glucose is high, while GDH1 mediated deamino-metabolism played a leading role on cells survival when glucose is insufficient, implying that major glutaminolysis pathways of HCC cells could change under different glucose concentration levels.

### GDH1 mediated glutamine anaplerosis facilitated the driven of TCA cycle in response to glucose limitation

**Fig 4:**
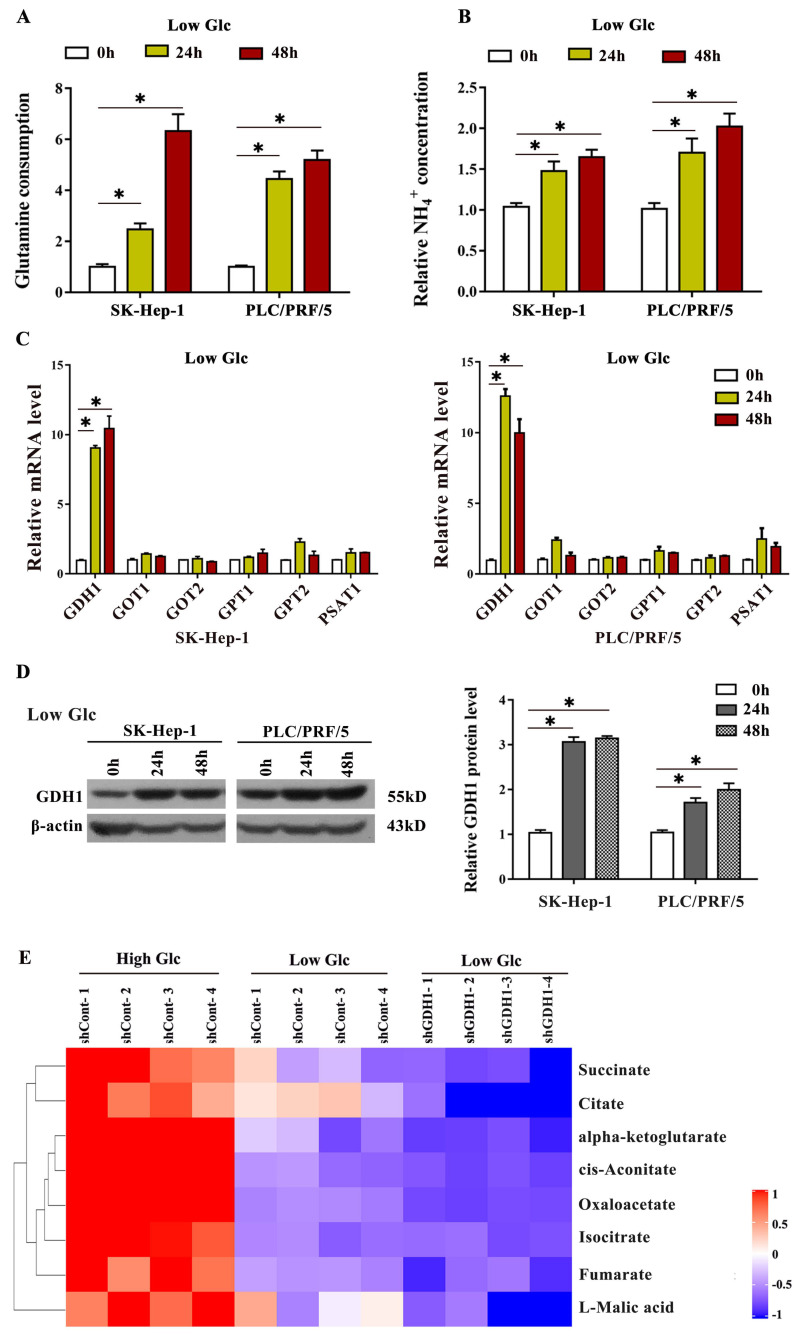
** GDH1 mediated glutamine anaplerosis prompted the driven of TCA cycle in response to glucose limitation**. **a**, Fold change in glutamine consumption in SK-Hep-1 and PLC/PRF/5 cells after cultivation in low-glucose medium (1.0 mM glucose) for 24 h and 48 h, respectively. *P < 0.05, when compared to 0 h group. **b,** Fold change in relative NH4^+^ concentration in HCC cells after cultivation in low-glucose medium (1.0 mM glucose) for 24 h and 48 h, respectively. *P < 0.05, when compared to the 0 h group. **c,** Enzymes mRNA levels in SK-Hep-1 and PLC/PRF/5 cells cultured in low glucose conditions were detected by qRT-PCR. *P < 0.05, vs. 0 h group. **d**, Enzymes protein levels in SK-Hep-1 and PLC/PRF/5 cells cultured in low glucose conditions were detected by Western blot. The signal intensities were quantified using Image J software. *P < 0.05, vs. 0 h group. **e**, Heat map comparing relative levels of TCA intermediates in GDH1-depleted PLC/PRF/5 cells after 3 days of low-glucose culture when compared with control (n = 4 independent samples per condition). Blue and red indicates down- or up-regulation, respectively.

In order to further confirm that glucose limitation promotes GDH1 mediated glutamine metabolic process, firstly, we assessed the glutamine consumption in HCC cells cultured in low-glucose medium. As shown in Fig. 4A, the levels of glutamine consumption in both two HCC cells were significantly elevated after incubation in glucose starvation condition for 24 h and 48 h. Secondly, we interrogated the levels of NH4+, a specific metabolite formed through a deamination reaction, under the same glucose limitation treatment. The results showed that NH4+ levels were obviously increased after glucose starvation (Fig. 4B). Additionally, we also found significant increase of GDH1 mRNA (Fig. 4C) and protein (Fig. 4D) levels on the basis of glucose limitation. Most importantly, our mass spectrometry results demonstrated that, compared to those in high glucose conditions, levels of TCA cycle intermediates including succinate, citrate, cis-aconitate, oxaloacetate and so on, were decreased in low glucose conditions, and the decrease of which were more significantly after silencing GDH1 expression (Fig. 4E). Together, these data provided evidences that GDH1 mediated glutamine anaplerosis facilitates the driven of TCA cycle for cell survival in response to glucose limitation.

### GDH1 expression was elevated in glucose-poor HCC tissues along with decreased expression of GOT1

**Fig 5:**
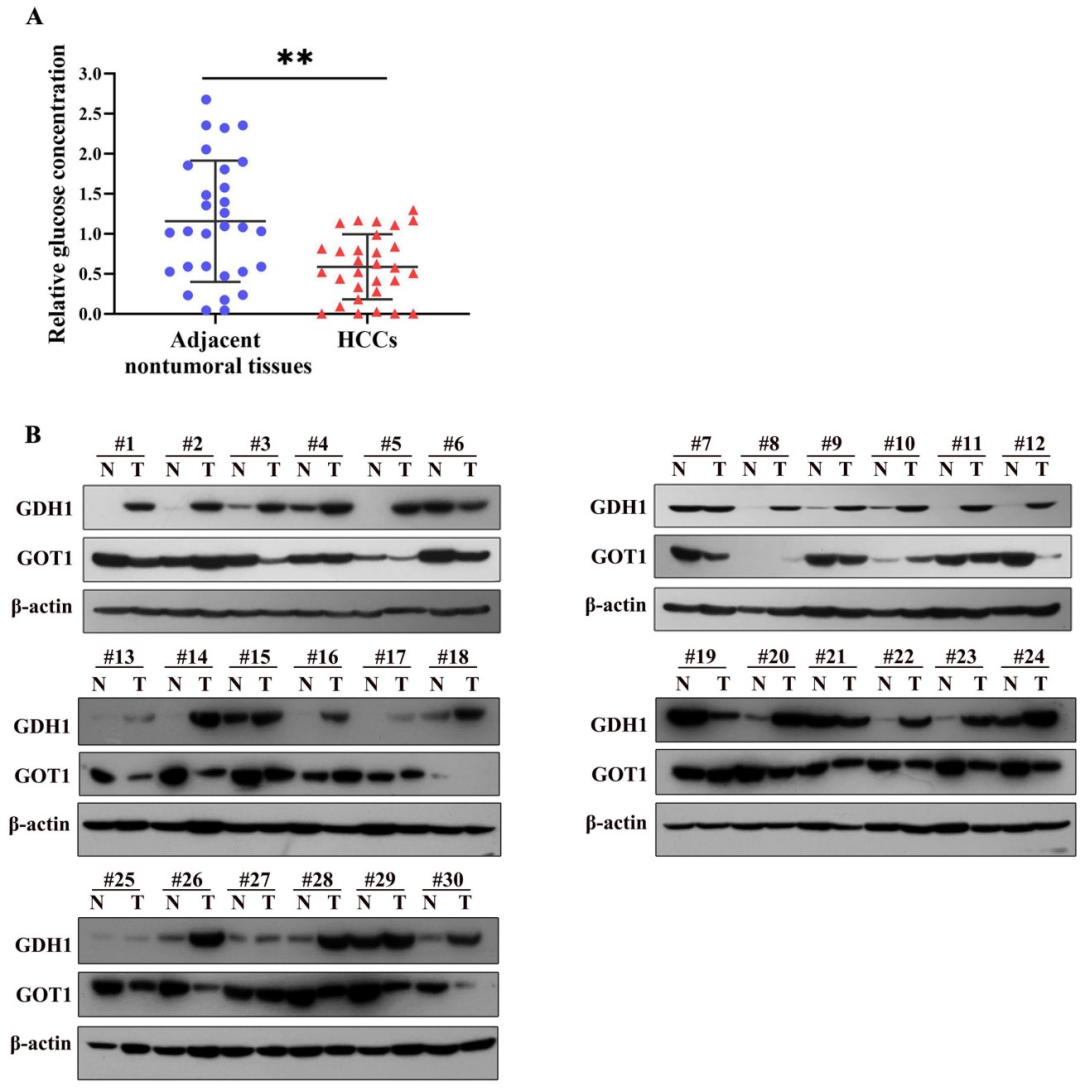
** GDH1 was upregulated in glucose-poor liver cancer tumor, accompanying with decreased GOT1**. **a**, Relative glucose levels in 30 paired clinical liver cancer tissues. The central line represents the mean and the error bars indicate the standard deviation. **P < 0.01, vs. adjacent nontumoral tissue group. **b**, Images of Western blot of GDH1 and GOT1 protein levels in glucose-poor liver cancer specimens.

Being a most consumed nutrient, glucose, are generally shown lower expression levels in tumor tissues than in non-tumor tissues 15. We detected glucose concentration in 30 paired HCC and adjacent nontumoral tissues, the results showed that glucose levels were strikingly decreased in HCC tissues compared to those in adjacent nontumoral tissue samples (Fig. 5A). In addition, we further examined the expression levels of GDH1 and GOT1 in above HCC tissues by western blotting. As shown in Fig. 5B, most protein levels of GDH1 were up-regulated in glucose-poor tumor tissues, simultaneously accompanied by decreased GOT1 protein levels, suggesting an underlying negative correlation between GDH1 and GOT1. But studies with larger sample size are still needed to definitively establish the relationship between GDH1 and GOT1 in HCC with low glucose concentrations.

## Discussion

Accumulating studies have found, in addition to glucose, that glutamine is also an important nutrient for tumor cells. Some tumor cells are more dependent on glutamine to maintain the energy requirements for their growth, exhibiting cell death follows Gln-deprivation, such phenomenon is defined as "*Gln addiction*" 16,17. Gln addiction has long been determined a characteristic of tumor cells. It has been shown that malignant cells such as glioma, lung cancer and kidney cancer were addicted to glutamine. In the present study, we confirmed that liver cancer cells strongly relied on glutamine uptake, which was in line with results of other researches 18,19. The inhibitory effect of Gln-deprivation on HCC cells growth is consistent with the results caused by glucose-deprivation. Being an important component of glutamine metabolism, glutaminolysis also plays a critical role in cancer cell metabolism, cell signaling, and cell growth in many cancers, which suggests a critical role of glutamine related downstream metabolites in tumor cells. Our experimental data further demonstrated the necessity of NEAAs and α-KG, two of the main metabolites of glutamine, on cell growth of HCC. Due to the whole process of glutaminolysis is an enzymatic reaction, these results indirectly suggest that some enzymes involved in glutaminolysis have play key roles in sustaining cell survival in HCC.

**Fig 6:**
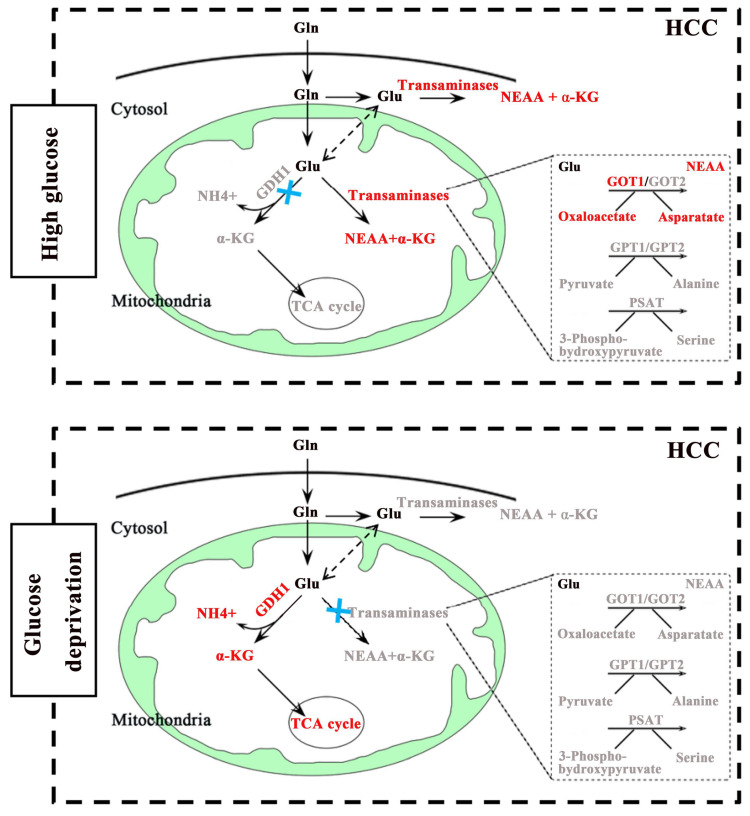
A schematic diagram of GDH1 and GOT1 mediated pathways in different glucose conditions in HCC.

Glucose and glutamine are the two main energy sources required for the rapid proliferation of tumor cells 20,21. Notably, the occurrence and development of some cancers seem to prefer aerobic glycolysis [22.23], while others are more dependent on glutaminolysis 24,25. Given the important role of glucose and glutamine in cell proliferation and survival, it is of great significance to clarify the relationship between glucose metabolism and glutaminolysis in HCC. To our knowledge, glutaminolysis is a highly coordinated process catalyzed by numerous enzymes. For example, GDH catalyzes glutaminolysis to form α-KG, which enters the TCA cycle. By contrast, transaminases including GOT, GPT and PSAT promote the generation of NEAAs to maintain cellular events. Therefore, the regulation of key metabolic enzymes appears particularly important in glutaminolysis. However, the interrelations between glutaminolysis-associated metabolic enzymes and glucose metabolism in cancers has not yet been exactly elucidated. In this study, our data showed that glutaminolysis was still warranted to maintain cell growth when glucose is sufficient, not to mention glucose starvation. Interestingly, our study validated that under different glucose concentrations, the glutaminolysis pathways which playing leading roles on cell survival were different. Specific manifestation was as follows: GOT1 mediated pathway played a dominant role in regulating HCC cells growth under high concentration of glucose conditions, yet was not activated when glucose was limited. However, GDH1 mediated enzymatic reaction was activated under glucose deprivation (Fig. 6). Surprisingly, we also uncovered a potential negative correlation between GDH1expression and GOT1expression in low-glucose HCC tissues, but our research didn't reveal any obviously regulatory effect of other aminotransferases, including GOT2, GPT1, GPT2 and PSAT1, on cell growth of HCC. These results highlighted the prominent places of GDH1 and GOT1 in the glutaminolysis process of HCC, suggesting that novel therapeutic approaches based on such two enzymes may be more beneficial to HCC treatment. Above all, the underlying opposing relationship between GDH1 and GOT1 supports the point that cancer cells adapt to nutrient-deprived tumor microenvironment during progression via adjusting the level and function of metabolic enzymes, that is, liver cancer cells maintain survival under different nutritional conditions through the metabolic flexibility of their glutamine-related enzymes. However, our data about the relationship between GDH1 and GOT1 in HCC is still preliminary. A more detailed analysis of the origin of this GOT1-GDH1 activity switch under glucose deprivation and of the importance of the GOT1-GDH1 switch in cell survival in vivo will be explored in our future studies.

It has been uncovered that glucose supplement and extra-cellular glucose concentration in tumor tissues are much lower than surrounding normal tissues 15, which indicated the irreplaceable role of GDH1 in the growth and survival of cancer cells, especially under low-glucose conditions. GDH1 is a key enzyme for glutaminolysis. Several studies have reported that GDH1 provides metabolic advantages for cancer cell proliferation and tumor metastasis via regulating the production of α-KG 26,27. Nevertheless, the pathological relationship between changes in GDH1 content and occurrence and development of HCC remains to be clarified in detail. Results of an earlier study by Jin et al. revealed that GDH1 predominantly controlled intracellular α-KG and subsequent fumarate levels, and contributed to redox homeostasis by activating GPx1, thereby promoting the cancer cell multiplying and tumor growth 28. Another published research showed that phosphorylated ELK1 activated by EGFR/MEK/ERK signaling pathway enriched in the promoter of GDH1 to stimulate the transcription of GDH1, then promoted glutamine metabolism 29. In the current study, we found an increased glutamine consumption in low-glucose cultured HCC cells, further demonstrated the necessity of glutaminolysis in HCC cells survival. It was reported that pyruvate carboxylase was highly expressed in glutamine-independent cancer cells, contributing to maintain anaplerosis under glutamine-deprivation conditions. In contrast, glutamine-dependent cell lines consume glutamine as the preferred anaplerotic substrate to drive TCA cycle 30. Results of our study found that NH4+, a specific metabolite produced in a reaction catalyzed by GDH1, was markedly elevated under glucose-limiting conditions, providing evidences that up-regulated GDH1 drives increased entry of glutamine-derived carbon into the TCA cycle in response to glucose starvation. Our finding concerning the driving role of GDH1 on TCA cycle under limited glucose status agreed well with the previously reported results, which further demonstrates a critical role for GDH1 in cell proliferation and tumor growth of HCC.

In conclusion, our study clarified a new insight of the regulation of glutaminolysis in HCC. That is, GDH1 mediated pathway played a leading role in maintaining cell survival under low glucose condition. By contrast, GOT1 mediated pathway was activated under high glucose condition. Moreover, highly expressed GDH1 could drive the TCA cycle in response to glucose deprivation. Our results also revealed a potential negative correlation between GDH1 and GOT1 in glucose-poor HCC tissues. These data enriched the understanding of metabolic flexibility in HCC, and provided certain theory guidance for the design and development of novel therapeutic methods targeting glutamine metabolism in HCC.

## Supplementary Material

Supplementary figures and tables.
