# Supplementary material for: Glutamate dehydrogenase 1 mediated glutaminolysis sustains HCC cells survival under glucose deprivation
Source: J Cancer. 2022 Jan 4;13(3):1061–72. doi: 10.7150/jca.64195 (PMC8824882; doi:10.7150/jca.64195)
Supplement: Supplementary file 1 — Supplementary figures and tables. [file jcav13p1061s1.pdf]

Supplementary data

Supplementary Figure S1

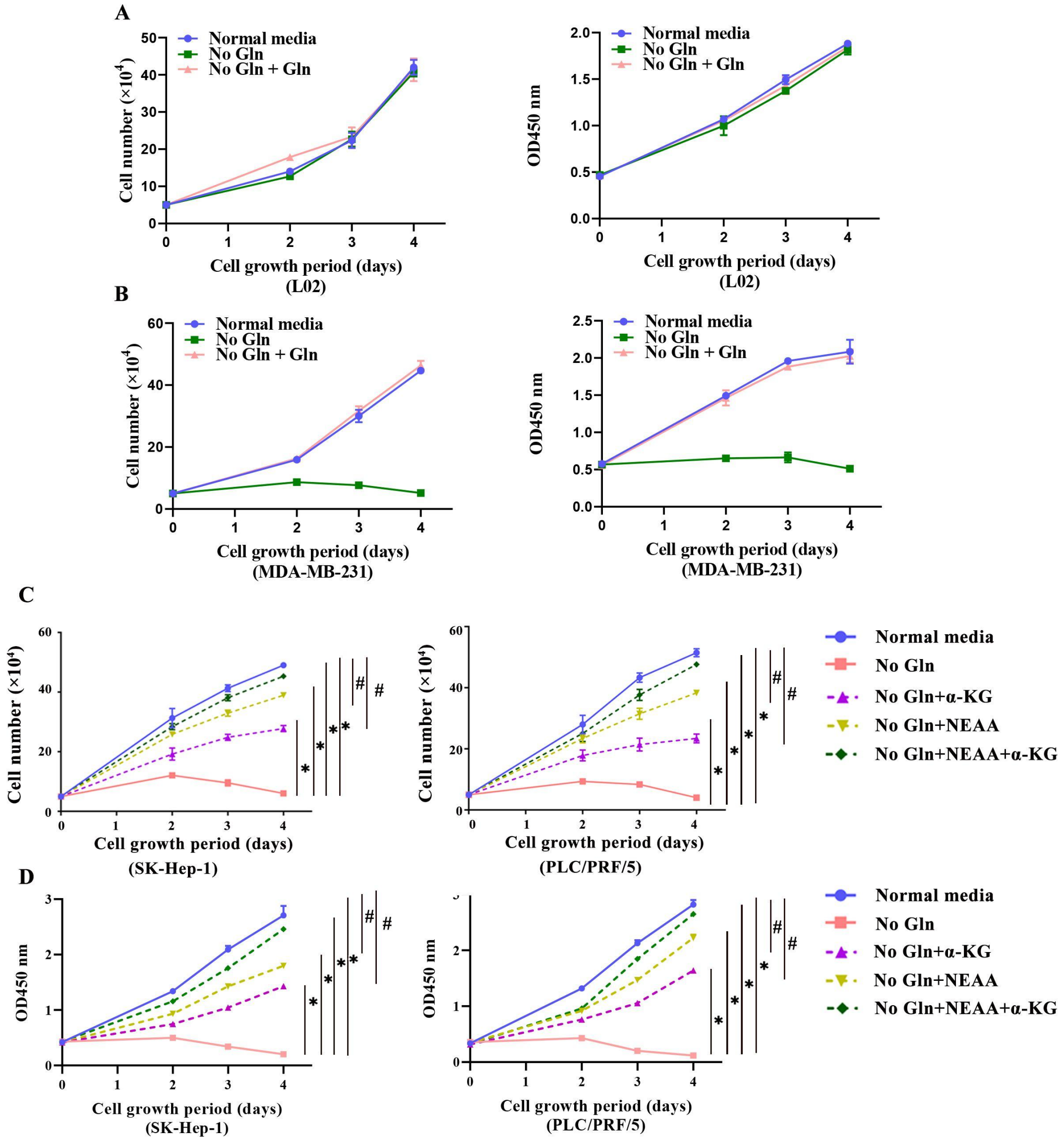

Supplementary Fig. S1 The growth dependency of cells on glutamine. a-b, Cells were cultured in normal medium (Normal media group), medium containing no glutamine (No Gln group) and glutamine-depleted medium additional supplement with 2mM glutamine (No Gln + Gln group), respectively. Normal liver cells L02 (**a**) and breast cancer cells MDA-MB-231 (**b**) were calculated at indicated time points using the trypan blue exclusion assay and CCK-8 assay, respectively. *$P < 0.05$, when compared to that in Normal media group. **c-d**, In addition to groups mentioned above, cells were cultured in medium supplemented with a-KG (2 mM) alone, nonessential

amino acid (NEAA, 0.1 mM glycine, alanine, aspartate, asparagine, proline and serine) alone, and a combination of a-KG and NEAA, respectively. Cells were calculated by the trypan blue exclusion assay (**c**) and CCK-8 assay (**d**), respectively. *$P < 0.05$, vs. No Gln group. #$P < 0.05$, vs. Normal media group

Supplementary Figure S2

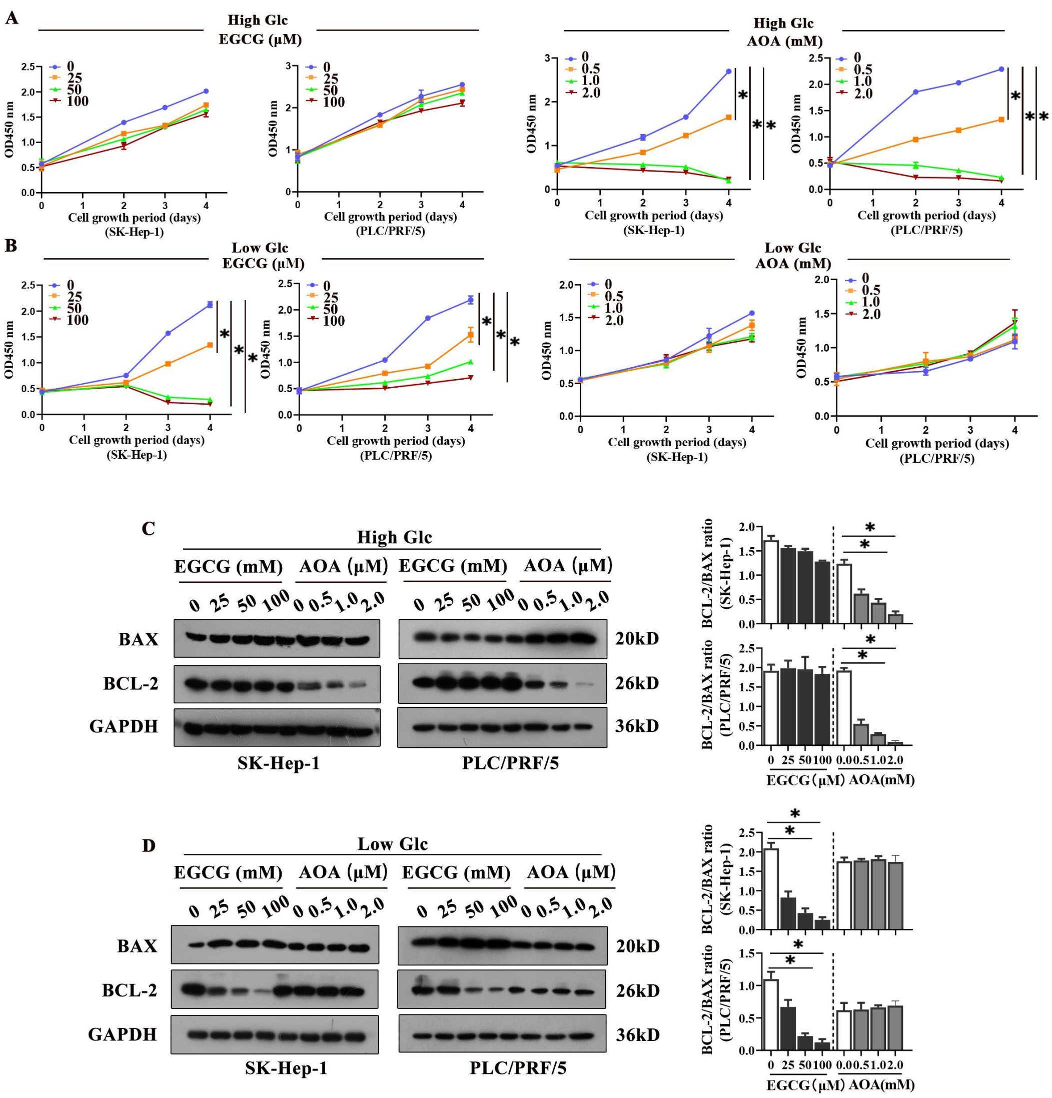

Supplementary Fig. S2 Major glutaminolysis pathway of HCC cells changed under different glucose concentration levels. a, SK-Hep-1 and PLC/PRF/5 cells were exposed to various concentrations of epigallocatechin gallate (EGCG) and aminooxyacetate (AOA) in high-glucose (25mM) conditions for 72 h, respectively. **b**, Similarly, cells were exposed to EGCG and AOA respectively and cultured in

low-glucose (1.0 mM) conditions for 72 h. The proliferation of cells were evaluated by CCK-8 assay. *$P < 0.05$, vs. 0 group. **c-d**, The levels of apoptotic markers BAX and BCL-2 were analyzed by Western blot after treatment with EGCG and AOA respectively under high (**c**) and low glucose (**d**) conditions for three days, respectively. The signal intensities were quantified by Image J software. *$P < 0.05$, vs. 0 group.

Supplementary Figure S3

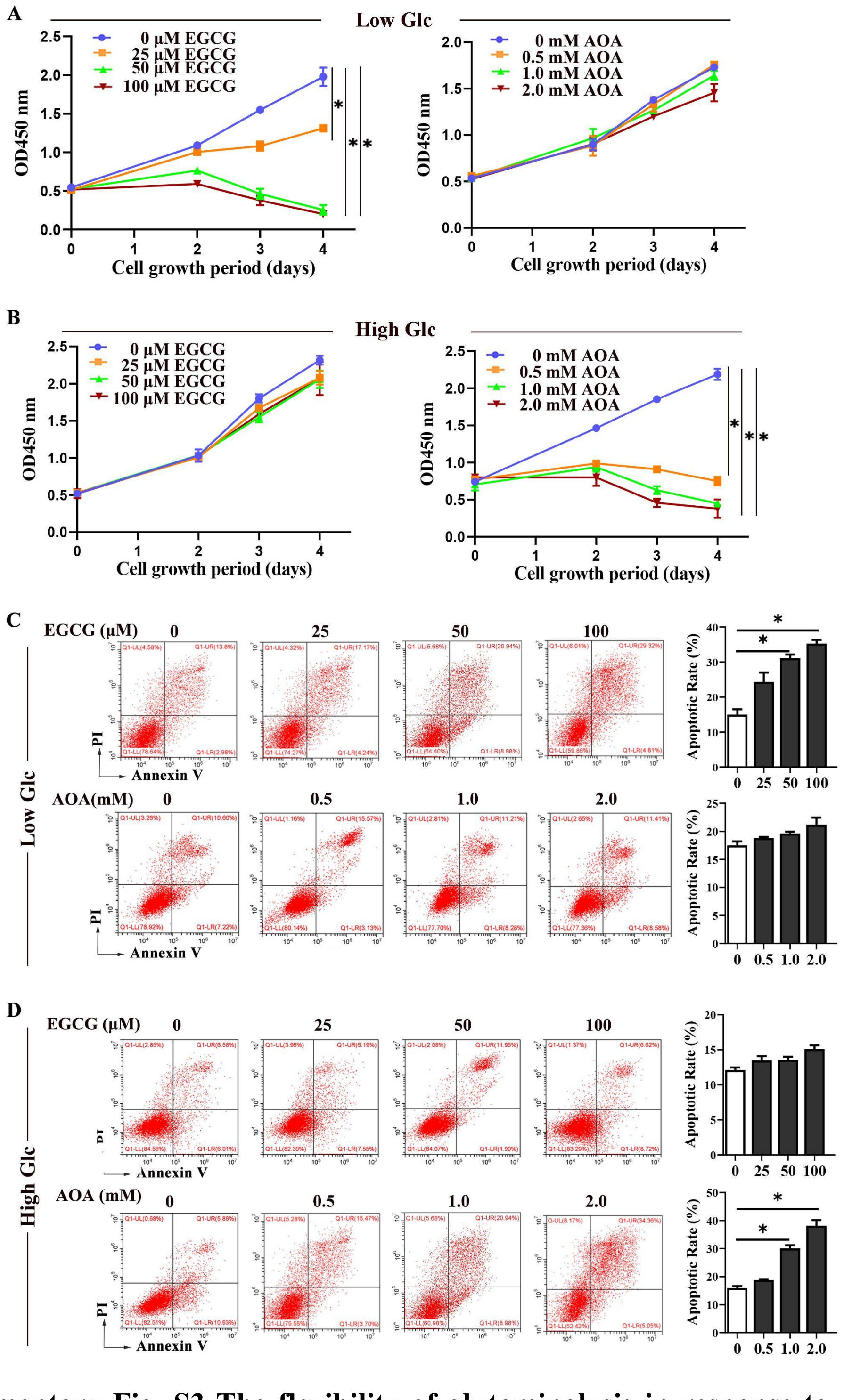

Supplementary Fig. S3 The flexibility of glutaminolysis in response to different glucose conditions in MDA-MB-231 cells. a, MDA-MB-231 cells were exposed to various concentrations of epigallocatechin gallate (EGCG) and aminooxyacetate (AOA) in low-glucose (1.0 mM) conditions for 72 h, respectively. **b**, Similarly, cells were exposed to EGCG and AOA respectively and cultured in high-glucose (25mM)

conditions for 72 h. The survival of cells were evaluated by CCK-8 assay. $*P < 0.05$, vs. 0 group. **c**, The apoptosis was analyzed by flow cytometer with Annexin V/PI after treatment with EGCG and AOA under low glucose conditions for three days, respectively. $*P < 0.05$, vs. 0 group. **d**, Similarly, the effect of various concentrations of EGCG and AOA on cells apoptosis was analyzed in high glucose media, respectively. $*P < 0.05$, vs. 0 group. Results were expressed as the average of three independent experiments (n=3 per group).

Supplementary Figure S4

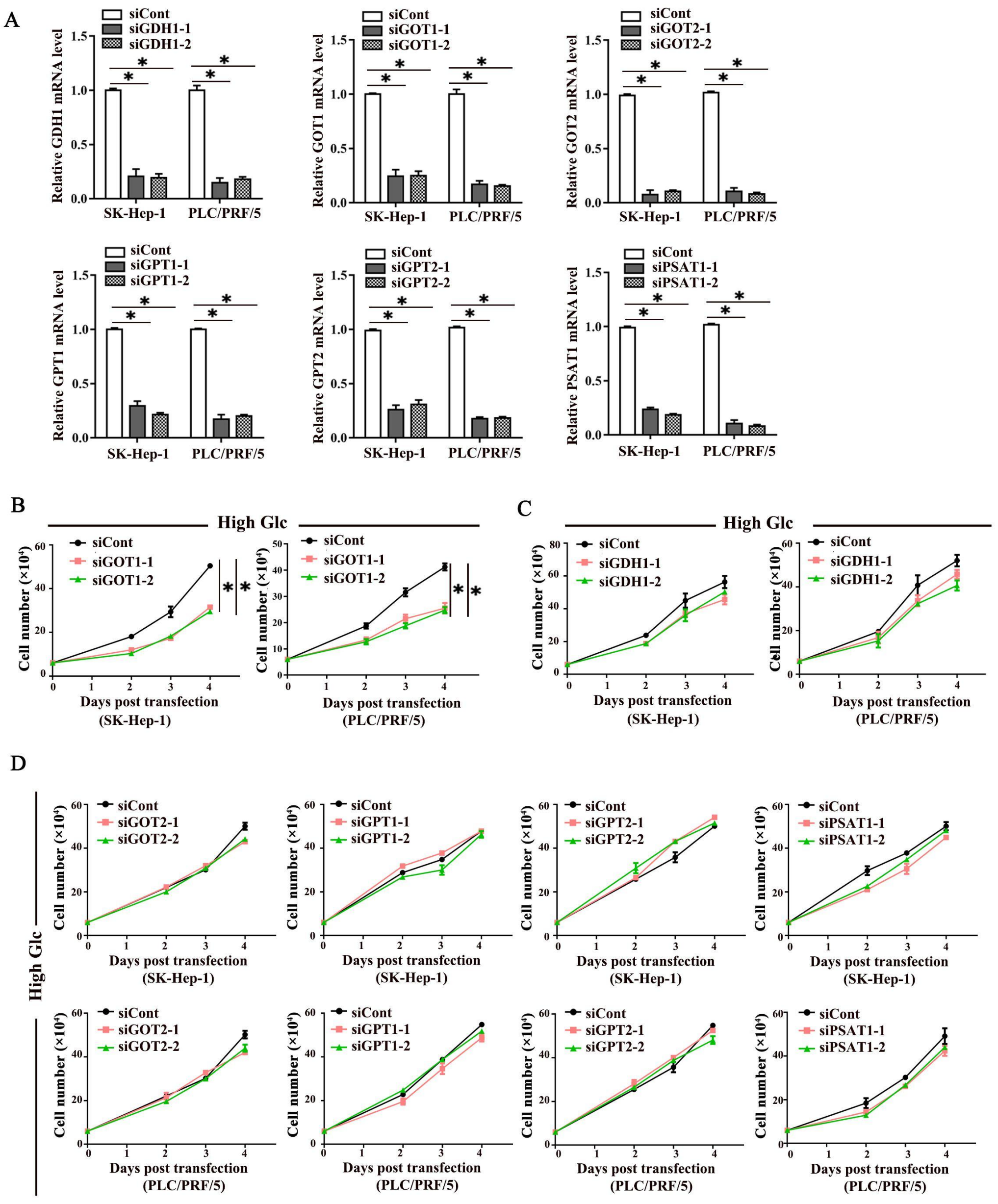

Supplementary Fig. S4 The effect of glutaminolysis enzymes knockdown on cell proliferation in liver cancer cells in high glucose. a, Inhibiting target genes by siRNA, the silence efficiency was demonstrated by qRT-PCR. *$P < 0.05$, vs. siCont. **b**, GOT1 silencing remarkably suppress the proliferation of SK-Hep-1 and PLC/PRF/5 cells as determined under high glucose levels. *$P < 0.05$, vs. siCont. **c**, GDH1 knock-down had no effect on proliferation of SK-Hep-1 and PLC/PRF/5 cells as determined under high glucose levels. **d**, Inhibition of Glu-dependent transaminases (GOT2, GPT1, GPT2, PSAT1) showed no significant effect on cell proliferation in SK-Hep-1 and PLC/PRF/5 cells under high glucose conditions.

Supplementary Figure S5

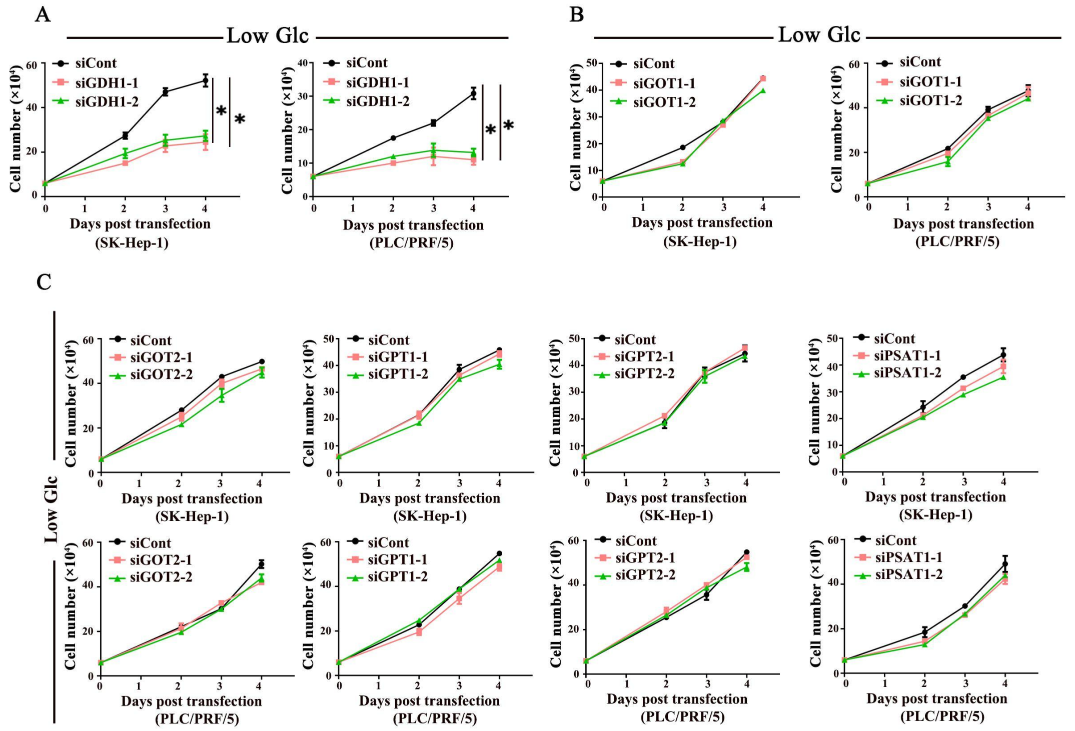

Supplementary Fig. S5 The effect of glutaminolysis enzymes knockdown on cell proliferation in liver cancer cells upon glucose limited. a, GDH1 silencing remarkably suppress the proliferation of SK-Hep-1 and PLC/PRF/5 cells as determined under low glucose levels. *$P < 0.05$, vs. siCont. **b**, GOT1 knock-down had no effect on proliferation of SK-Hep-1 and PLC/PRF/5 cells as determined under low glucose levels. *$P < 0.05$, vs. siCont. **c**, Inhibition of Glu-dependent

transaminases (GOT1, GOT2, GPT1, GPT2, PSAT1) showed no significant effect on cell proliferation in 1.0mM glucose condition.

Supplementary Table S1

Primers of qPCR used in this study

Primer names	Sequences
GDH1 sense	5'TTGGTCCCGGTGTCTGTGTC3'
GDH1 anti-sense	5'AACGGCACATCAACCACTGC3'
GOT1 sense	5'AGCTGTGCTTCTCGTCTTGC3'
GOT1 anti-sense	5'CCCAAAGATTGCACACCTCC3'
GOT2 sense	5'TGACATGGCCTACCAAGGCT3'
GOT2 anti-sense	5'GGCTCCTACACGCTCACCAT3'
GPT1 sense	5'GGGTTCGCAGTTCCACTCATT3'
GPT1 anti-sense	5'CCGCACACTCATCAGCTTCA3'
GPT2 sense	5'CAGGAGGGATGGCGGTGTG 3'
GPT2 anti-sense	5'CACACCTGTCCGTGACTTGC 3'
PSAT1 sense	5'TGGTCAACTTTGGGCCTGGT3'
PSAT1 anti-sense	5'CAGCTAGCAATTCCCGCACA3'
β-actin sense	5'CTCTTCCAGCCTTCCTTCCT3'
β-actin anti-sense	5' AGCACTGTGTTGGCGTACAG3'

Supplementary Table S2

Targeting sequence of siRNAs used in this study

Primer names	Sequences
siGDH1-1	5’ GCACCTGCGGATCATCAA 3’
siGDH1-2	5’ GCGTTCTGCCAGGCAAATTTT 3’
siGOT1-1	5’ GCGTTGGTACAATGGAACAAA 3’
siGOT1-1	5’ GCTAATGACAATAGCCTAAAT 3’
siGOT2-1	5’ GGAATCTCTTTGCGTTCTTTG 3’
siGOT2-2	5’ CTTTAAGAGGGACACCAATCTC 3’
siGPT1-1	5’CCACTTCCGGATGACCATTTT 3’
siGPT1-2	5’GCAGGTGGATTACTACCTGTT 3’
SiGPT2-1	5’ CGGCATTTCTACGATCCTGAA 3’
SiGPT2-2	5’ CCATCAAATGGCTCCAGACAT 3’
siPSAT1-1	5’ GCCAAGAAGTTTGGGACTATA 3’
siPSAT1-2	5’ CCAGACAACTATAAGGTGATT 3’
siCont	5’TTCTCCGAACGTACGTTT 3’
